# The Transcriptomic Landscape and Regulatory Signaling Features of Bovine Skeletal Muscle Cells Used for Cultured Meat Production

**DOI:** 10.3390/foods15061074

**Published:** 2026-03-19

**Authors:** Xing Zhen, Se-Hee Choe, Eun Young Kim, Yingying Mao, Ryoung Eun Kim, Jae-Won Huh, Min Kyu Kim, Jong-Hee Lee

**Affiliations:** 1 National Primate Research Center (NPRC), Korea Research Institute of Bioscience and Biotechnology (KRIBB), Cheongju 28116, Republic of Korea; zhenxing93@kribb.re.kr (X.Z.); csh91@kribb.re.kr (S.-H.C.); maoyingying@o.cnu.ac.kr (Y.M.); 2 Department of Nanoscience and Nanotechnology, Graduate School, Kyungpook National University, Daegu 41566, Republic of Korea; 3 MK Biotech Inc., Daejeon 34134, Republic of Korea; key@mkbiotech.co.kr (E.Y.K.) kre@mkbiotech.co.kr (R.E.K.); 4 Division of Animal and Dairy Science, College of Agriculture and Life Science, Chungnam National University, Daejeon 34134, Republic of Korea; 5 Department of Advanced Bioconvergence, KRIBB School of Bioscience, University of Science and Technology (UST), Daejeon 34113, Republic of Korea

**Keywords:** cultured meat, bovine muscle stem cells, transcriptomics, autophagy, myogenesis

## Abstract

Cultured meat, a sustainable alternative to conventional meat, addresses ethical and environmental challenges in livestock production. Its production relies on bovine muscle stem cells from adult muscle or fetal tissue, whose proliferation and differentiation vary with age and developmental stage. However, the molecular mechanisms underlying these variations remain unclear. RNA sequencing was performed to characterize the transcriptomic landscape of bovine muscle stem cells across developmental stages, including myogenic maturation. Differentially expressed genes and key signaling pathways regulating myogenesis were identified, and the functional impact of modulating the AKT-autophagy pathway on differentiation was assessed. Transcriptomic analysis revealed distinct age-dependent gene expression patterns. It was possible to classify cells into three categories: young undifferentiated, young differentiated, and old differentiated. Young undifferentiated-like cells exhibited upregulation of genes associated with active states during the transitions from quiescence to activation and, ultimately, to commitment, indicating that they had robust differentiation potential. In contrast, aged myogenic samples displayed gene expression profiles that acted as barriers to efficient myogenic differentiation. Notably, modulation of the AKT-autophagy pathway both facilitated the production of very mature myogenic cells and prevented spontaneous differentiation, thereby preserving differentiation capacity in vitro. These findings provide insights into age-dependent muscle stem cell differentiation and suggest strategies to optimize cultured meat production. The appropriate modulation of key signaling pathways may help us to overcome major challenges in achieving scalable and efficient cultured meat manufacturing.

## 1. Introduction

Cultured meat, as an emerging technology, has attracted considerable attention and has been proposed as an approach to address the environmental, ethical, and food security challenges associated with conventional livestock production [1]. This innovative approach involves the in vitro culture of animal-derived cells, particularly muscle stem cells, and is being explored as a potential strategy for producing meat products that may contribute to more sustainable food systems and help address the increasing global demand for animal protein [2]. The foundation of cultured meat production is the efficient in vitro differentiation and proliferation of muscle stem cells, primarily sourced from animals such as bovines [3]. These cells, commonly termed muscle satellite cells, along with other supporting cells, serve as the primary building blocks for cultured skeletal muscle tissue [4].

Although tissue engineering techniques have greatly improved the isolation, expansion, and myogenic differentiation of muscle stem cells [5], the efficiency of cultured meat production remains influenced by biological factors such as donor age, breed, and tissue origin, which can affect cell growth, differentiation potential, and production scalability [6,7]. Moreover, during in vitro culture, it remains unclear to what extent muscle stem cells can recapitulate the complex physiological behaviors and cellular interactions observed in vivo, as the molecular mechanisms governing muscle stem cell differentiation are still not fully elucidated. No logical strategies that might enhance differentiation efficiency are currently known [8]. Understanding these mechanisms is therefore essential for optimizing primary cell expansion and improving cultured meat production [9]. Previous studies have extensively investigated skeletal muscle composition, differentiation pathways, and developmental mechanisms in humans and rodents, providing important insights into muscle biology [10,11,12]. However, comparable knowledge for agriculturally important species such as bovines remains limited. In particular, comprehensive transcriptomic analyses of fetal muscle stem cells across different stages of bovine muscle development are scarce, hindering the optimization of cell culture strategies and the improvement of cultured meat production efficiency.

Muscle stem cells, also termed satellite cells, are quiescent under homeostatic conditions and reside between the basal lamina and sarcolemma of muscle fibers [13]. Upon muscle injury or stimulation by specific signaling pathways, these cells become activated and enter proliferation and differentiation programs. They first form myoblasts, which subsequently fuse and differentiate into mature myotubes or myofibers, thereby facilitating muscle repair and regeneration [14]. This regenerative process is regulated by several key signaling pathways, which collectively determine whether cells proliferate, differentiate, or return to quiescence, thereby maintaining the regenerative capacity of skeletal muscle [15,16]. Among these mechanisms, autophagy plays an important role as a tightly regulated cellular degradation pathway that maintains cellular homeostasis and adaptation to environmental changes. In muscle stem cells, autophagy contributes to multiple stages of muscle homeostasis and regeneration, including quiescence, activation, and differentiation [17,18]. Basal autophagy operates continuously in quiescent stem cells to preserve cellular integrity and stemness, while its upregulation during activation supports the increased bioenergetic demands of proliferating cells. Properly regulated autophagy is also required for myoblast differentiation and myotube formation; dysregulation of autophagy, whether excessive or insufficient, can impair differentiation and compromise muscle regeneration [19], highlighting the importance of balanced autophagic activity throughout the myogenic process.

In this study, RNA sequencing was utilized to investigate transcriptomic changes in bovine skeletal muscle cells across different developmental stages. This approach revealed that sample age and maturation conditions significantly influenced the activation or suppression of various signaling pathways. Among these, modulation of the AKT-autophagy axis emerged as a key strategy when seeking to enhance differentiation efficiency. By fine-tuning this pathway, optimized protocols were established that yielded high-purity muscle cells with minimal unwanted spontaneous differentiation during in vitro culture. These findings provide valuable insights into age-related variations in muscle cell behavior and may contribute to the ongoing efforts to develop more efficient and scalable cultured meat production strategies.

## 2. Materials and Methods

### 2.1. Cell Culture and Differentiation

Fresh skeletal muscle tissues were collected from cattle (*Bos taurus*) at different developmental stages. The breed used was Korean native cattle (Hanwoo). The Young group consisted of three bovine fetuses (Young #1–#3, all male), whereas the Old group consisted of three adult cattle (Old #1–#2, male; Old #3, female). All samples were collected from the top round muscle of the hindlimb to minimize variation arising from different muscle groups. The sample identification numbers (#1–#3) represent independent biological samples from different animals. All Old males were intact (not castrated) adult cattle. Detailed sample information, including age category and annotations of the sample IDs, is provided in Figure S1A. The collected tissues were immediately washed with PBS (welgene, Gyeongsan-si, Republic of Korea, CAT: LB001-02). Each tissue sample was then cut into small pieces approximately 1–2 mm^3^ in size and fully immersed in a digestion solution containing 0.25% trypsin (Gibco, Grand Island, NY, USA, CAT: 25200056) and 1 mg/mL collagenase (Gibco, CAT: 17018029). The tissue was incubated at 37 °C for 30–45 min with gentle rocking every 10 min to ensure thorough digestion. After digestion, the mixture was filtered through a 100 µm cell strainer. The resulting cell suspension was centrifuged at low speed (300 g, 5 min) to maintain viability for subsequent cell culture. The collected cells were resuspended in Skeletal Muscle Cell Growth Medium (PromoCell, Heidelberg, Germany, CAT: C-23060). To promote myocyte differentiation, cells were cultured to 80–90% confluence, after which the growth medium was replaced with Skeletal Muscle Cell Differentiation Medium (PromoCell, CAT: C-23061). The medium was refreshed every 48 h over a 6-day culture period, during which the cells formed multinucleated myotubes. C2C12 mouse myoblasts (ATCC) were cultured in DMEM medium (Gibco, CAT: 11965092) supplemented with 10% fetal bovine serum (FBS, Gibco, CAT: 16000044) and 1% penicillin-streptomycin (Gibco, CAT: 15140122). To induce muscle differentiation, these cells were transferred into fresh medium after digestion with 0.25% trypsin. When the cells reached 80–90% confluence, the medium was replaced with DMEM supplemented with 1% FBS and changed every 48 h over 4 days to promote differentiation.

### 2.2. RNA Extraction and Sequencing

RNAs were purified using RNeasy Mini Kits (Qiagen, Venlo, The Netherlands, CAT: 74104) and dissolved in RNase-free water [20]. RNA quantity and quality were assessed using a Bioanalyzer 2100 (Agilent Technologies, Santa Clara, CA, USA) and a Nanodrop device (Thermo, Waltham, MA, USA), respectively. Total RNA with RNA integrity numbers (RINs) >8 was used to prepare cDNA libraries using the TruSeq Stranded Total RNA Kit (Illumina, San Diego, CA, USA, CAT: 20040526) following the manufacturer’s protocol. Libraries were sequenced as 101 bp paired-end reads on an Illumina NovaSeq 6000 platform.

### 2.3. Expression Analysis

Clean reads were obtained from the raw sequencing data by discarding adapter sequences from the Illumina TruSeq kit (Illumina, CAT: 20020594), removing low-quality reads, and filtering out rRNA sequences. The clean reads were then mapped to the ARS-UCD1.2 *Bos taurus* genome. Gene expression levels were quantified using the “fragments per kilobase of transcript per million mapped reads” (FPKM) method implemented in StringTie version 2.1.3b [21]. The resulting expression values were normalized using DESeq2 v1.38.3 [22] and subjected to principal component analysis (PCA) using the prcomp function in R v4.2.3. A hierarchical clustering heatmap was generated using the pheatmap package v1.0.12. Clusters and dendrograms were constructed using the hclust function and ggdendro package v0.1.23, respectively [23].

### 2.4. Differentially Expressed Genes

Differentially expressed genes (DEGs) were identified via pairwise comparisons using DESeq2 v1.38.3 [22]. Genes were considered differentially expressed when the *p*-value of the comparison was <0.05, and the log_2_-fold change was greater than 2 or less than −2.

### 2.5. Functional Annotation

Gene ontology (GO) and Kyoto Encyclopedia of Genes and Genomes (KEGG) annotations were performed using clusterProfiler v4.6.2 [24]. This software supports statistical analysis and visualization of the functional profiles of genes and gene clusters.

### 2.6. Hub Gene Identification

The STRING v2.0.0 tool was used to construct the protein–protein interaction network. Cytoscape v3.9.1 [25] was used for network visualization. The confidence score was set to 0.4, and the “maximum additional interactors” parameter (default) was used. Within the protein–protein interaction network, a subnetwork of hub genes was identified using the cytoHubba v0.1 plugin [26] in Cytoscape. Hub gene selection was based on the Maximal Clique Centrality (MCC) algorithm. The 20 genes with the highest MCC values were defined as hub genes.

### 2.7. Immunofluorescence Assay

Immunofluorescence staining was performed as previously described [27,28]. Cells were fixed and permeabilized using a fixation/permeabilization solution (BD Biosciences, Franklin Lakes, NJ, USA). To block non-specific binding, cells were incubated in 1× Wash/Perm buffer (BD Biosciences, CAT: 554723) at room temperature for 20 min. Primary antibodies (Table S2) were then applied, followed by overnight incubation at 4 °C. After washing, cells were incubated with fluorescently conjugated secondary antibodies at room temperature for 1 h in the dark. Nuclei were stained with Hoechst 33342 (Invitrogen, Carlsbad, CA, USA, CAT: R37605), and fluorescent signals were visualized under a Zeiss fluorescence microscope (Oberkochen, Germany).

### 2.8. Western Blotting

Cells were lysed on ice using a protein extraction buffer containing a protease inhibitor cocktail (Sigma, Hong Kong, China, CAT: P8340). Protein concentration was determined using the Pierce BCA Protein Assay Kit (Thermo, Waltham, MA, USA, CAT: 23227). Equal amounts of protein were subjected to 10% SDS-PAGE and transferred to nitrocellulose membranes (Bio-Rad, Hercules, CA, USA, CAT: 1620115). To block non-specific binding, membranes were incubated with EveryBlot Blocking Buffer (Bio-Rad, CAT: 12010020) for 30 min at room temperature and then probed with primary antibodies (Table S2) overnight at 4 °C. After washing with 1× TBST (LPS, Hong Kong, China, CAT: CBT007L), membranes were incubated with HRP-conjugated secondary antibodies for 4 h at room temperature. Immunoreactive bands were visualized using the developer/fixer film system as previously described [29].

### 2.9. RT-qPCR

Total cellular RNA was extracted using the standard protocol of the RNeasy Mini Kit. cDNA synthesis was performed using the ReverTra Ace-α^®^ High-Efficient Reverse Transcription Kit (Toyobo, Osaka, Japan, CAT: FSK-101F). Real-time quantitative PCR (RT-qPCR) was conducted using the TB Green Premix (TaKaRa, San Jose, CA, USA, CAT: RR820A) system and specific primers (Table S1). A StepOne Real-Time PCR System (Applied Biosystems, Waltham, MA, USA) was used for amplification.

### 2.10. Statistics

Quantitative data are expressed as means ± standard errors of the mean (SEMs). All statistical analyses were conducted using GraphPad Prism v8.0. The two-tailed *t*-test was used to compare two groups, whereas one-way analysis of variance with multiple comparisons was utilized for comparisons among multiple groups. The threshold for statistical significance was defined as *p* < 0.05. Unless otherwise noted in figure legends, all experiments were performed in triplicate.

## 3. Results

### 3.1. Transcriptome Analysis of Differentially Expressed Genes During Myoblast Differentiation

**Figure 1:**
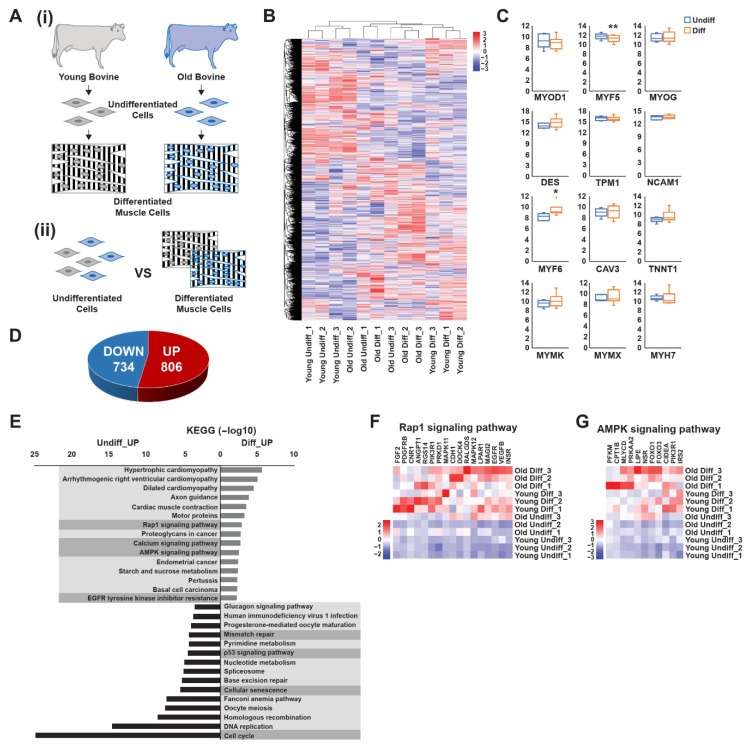
Transcriptome analysis of differentially expressed genes during myoblast differentiation of bovine muscle stem cells. (**A**) Schematic overview of the experimental design showing undifferentiated and differentiated bovine muscle stem cells used for transcriptomic analysis. Primary bovine muscle stem cells were isolated from the top round skeletal muscle of the bovine hindlimb collected at different developmental stages, including three fetal samples (Young #1–#3, all male) and three adult cattle (Old #1, 75 months; Old #2, 52 months; Old #3, 58 months; Old #1–#2 male, Old #3 female). To induce myogenic differentiation, cells were cultured to 80–90% confluence and then switched to skeletal muscle cell differentiation medium. The medium was refreshed every 48 h for 6 days, during which multinucleated myotubes were formed, (i) isolation and culture of primary bovine muscle stem cells derived from young and old donors, (ii) preparation and comparative analysis of RNA sequencing samples from undifferentiated and differentiated bovine muscle stem cells. (**B**) Heatmap showing the most prominent differentially expressed genes among young undifferentiated, old undifferentiated, young differentiated, and old differentiated cells. Genes (rows) and samples (columns) are clustered by fold changes from −3 to 3. (**C**) Median fold expression levels of selected prominent myogenic transcription factors and canonical differentiation markers in undifferentiated (Young and Old) and differentiated (Young and Old) cells, each normalized to the levels in undifferentiated cells, as determined via RNA sequencing. (**D**) Pie chart displaying the numbers of genes that exhibited the greatest upregulation and downregulation differences in differentiated cells, each normalized to the levels in undifferentiated cells. (**E**) Bar plot showing KEGG pathway enrichments corresponding to genes differentially expressed by undifferentiated and differentiated cells. The proportions of significantly upregulated genes (of all genes) for each KEGG pathway are shown. The *x*-axis represents the statistical significance expressed as −log10 (*p* value), and the *y*-axis indicates the pathways. (**F**) Heatmap showing the most differentially expressed genes of the Rap1 signaling pathway between young undifferentiated, old undifferentiated, young differentiated, and old differentiated cells. Genes (columns) and samples (rows) are clustered by fold changes from −2 to 2. (**G**) Heatmap showing the most differentially expressed genes of the AMPK signaling pathway between young undifferentiated, old undifferentiated, young differentiated, and old differentiated cells. Genes (columns) and samples (rows) are clustered by fold changes from −3 to 3. Boxplot data are the first quartile, median, and third quartile. Values are means ± SEMs. * *p* < 0.05, ** *p* < 0.01.

To investigate transcriptional changes during myoblast differentiation, we identified DEGs between undifferentiated and differentiated muscle cells across all samples (Figure 1A and Figure S1A). A total of 1540 DEGs (DEG set 1) were identified in differentiated cells compared with undifferentiated cells, including 806 upregulated and 734 downregulated genes (Figure 1D). Hierarchical clustering analysis revealed that young bovine samples exhibited distinct transcriptional shifts after differentiation, whereas gene expression changes in older samples were comparatively less pronounced. Additionally, inter-individual variability was observed among the differentiated older samples; however, this variability did not appear to be associated with sex, as the female Diff-sample (Old #3) clustered closely with Diff-sample Old #2, while the male Diff-sample (Old #1) showed greater divergence from the other two samples (Figure 1B,C). To further characterize these transcriptional changes, the upregulated and downregulated DEGs were subjected to separate KEGG pathway and GO enrichment analyses. KEGG pathway analysis revealed that several signaling pathways were significantly enriched among the differentially expressed genes. Among these, pathways with established roles in muscle differentiation and energy metabolism were of particular interest, including Rap1 signaling, calcium signaling, and AMPK signaling. In addition, upregulated genes were also enriched in the EGFR tyrosine kinase inhibitor resistance pathway (Figure 1E). Conversely, downregulated DEGs were primarily associated with mismatch repair, p53 signaling, cellular senescence, and cell cycle pathways (Figure 1E). Based on their known roles in muscle differentiation and energy metabolism, Rap1 and AMPK signaling pathways were selected for further analysis [30,31]. Genes associated with the Rap1 and AMPK signaling pathways were markedly upregulated in differentiated muscle cells compared with undifferentiated cells (Figure 1F,G and Figure S1B,C). These pathways are known to play important roles in myoblast differentiation and metabolic regulation, suggesting their involvement in promoting muscle cell maturation and maintaining muscle cell function. In contrast, genes associated with mismatch repair, p53 signaling, cellular senescence, and the cell cycle were more highly expressed in undifferentiated cells (Figure 1E and Figure S1F–I), suggesting that these pathways actively maintain the proliferative state of myoblasts before differentiation. GO analysis further illuminated biological processes (BP) and molecular functions (MF) influenced by myoblast differentiation (Figure S1D,E). Upregulated genes were enriched in muscle system processes, muscle contraction, and muscle development pathways, confirming their essential roles in skeletal muscle differentiation. Conversely, genes related to DNA replication, chromosomal organization, cell division, and the cell cycle were enriched in undifferentiated cells, supporting their involvement in maintaining proliferative capacity. Enriched molecular functions in undifferentiated cells included 3′,5′-cyclic-AMP phosphodiesterase activity, signaling receptor activity, and transmembrane signaling receptor activity. In contrast, several signaling-related components, including transmembrane receptors such as PDGFR, EGFR, and INSR, showed increased abundance in differentiated cells. The observed differences in gene expression dynamics between undifferentiated and differentiated states further suggest that cell cycle regulation and senescence-associated pathways play central roles in controlling differentiation potential.

### 3.2. Global Transcriptomic Analysis of Bovine Myoblast Differentiation

**Figure 2:**
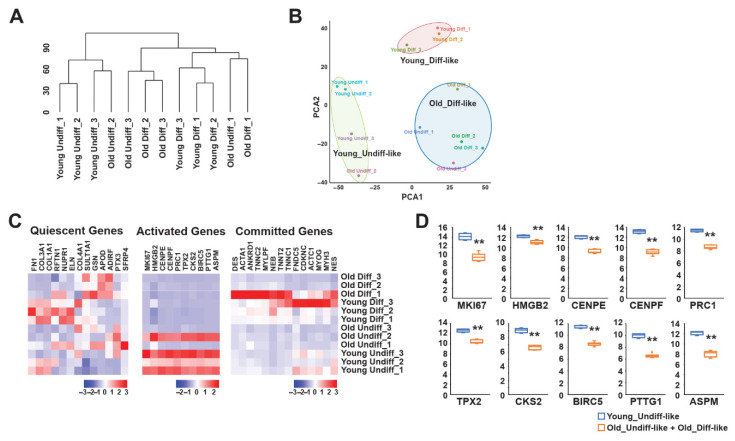
Comprehensive transcriptome profiling during bovine myoblast differentiation. (**A**) Hierarchical clustering identified correlations among young undifferentiated, old undifferentiated, young differentiated, and old differentiated cells. (**B**) Principal component analysis (PCA) of cell types revealed “young-undiff-like,” “young-diff-like,” and “old-diff-like” clusters. (**C**) Heatmap showing the most differentially expressed genes among quiescent, activated, and committed genes across young undifferentiated, old undifferentiated, young differentiated, and old differentiated cells. Genes (columns) and samples (rows) are clustered by fold changes from −3 to 3 or −2 to 2. (**D**) Expression levels of selected activation-related genes in the young-undiff-like, old-undiff-like, and old-diff-like clusters, as determined via RNA sequencing. Boxplot data are the first quartile, median, and third quartile. Values are means ± SEMs. ** *p* < 0.01.

We assessed the global transcriptomic landscape of bovine myoblast differentiation. Hierarchical clustering and PCA were conducted to explore sample distributions and similarities. The PCA plot reveals that young bovine samples segregated more distinctly than old samples, whose differentiation status appeared less defined. We categorized the samples into three broad transcriptional clusters: Young-Undiff-like, Young-Diff-like, and Old-Diff-like. The validity of this classification was partially supported by hierarchical clustering, which suggested age-related differences in transcriptional regulation during myogenic differentiation (Figure 2A,B). A recent single-cell transcriptomic study on bovine muscle-derived cell types used for cultured meat production reported three subpopulations—quiescent, activated, and committed—during developmental transitions [32]. In partial agreement with these findings, our bulk transcriptomic analysis revealed a decline in activated gene expression in differentiated samples relative to their undifferentiated counterparts (Figure 2C). However, this trend was not entirely consistent within the old bovine group, where only the “Old Undiff-2” sample exhibited a distinct reduction in activation. A comparison of gene expression patterns across the PCA-defined clusters revealed that gene activation was most prevalent in undifferentiated samples, particularly those from young bovines (Figure 2C,D). These findings suggest that undifferentiated muscle cells from young bovine samples are both active and primed for differentiation, whereas aging may impair the transcriptional activation necessary for efficient myogenesis.

### 3.3. Age-Specific Characterization of Undifferentiated Myoblasts

**Figure 3:**
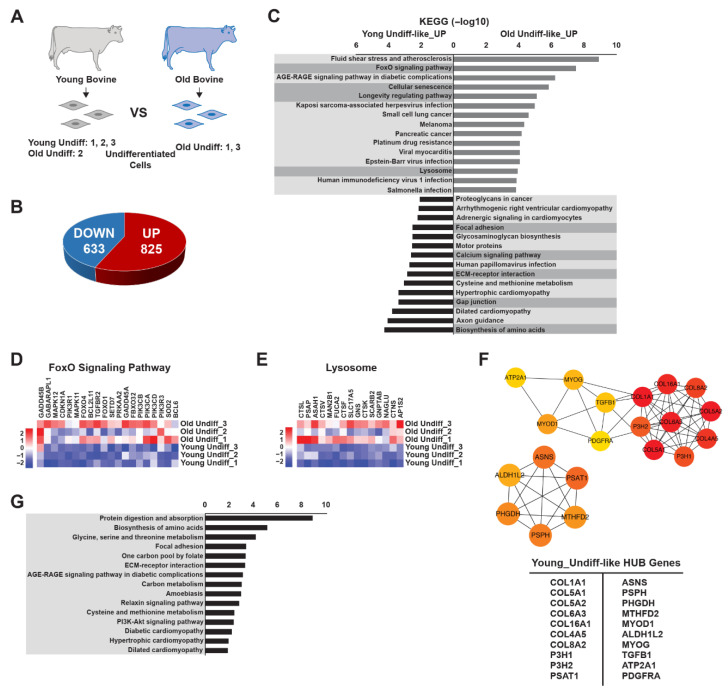
Age-dependent profiling of undifferentiated myoblasts. (**A**) Schematic representation of young and old undifferentiated bovine muscle stem cells used for transcriptome analysis. (**B**) Pie chart displaying the numbers of genes showing the greatest differences in upregulation and downregulation between the young-undiff-like and old-undiff-like clusters. (**C**) Bar plot showing KEGG pathway enrichments of genes differentially expressed in the young-undiff-like and old-undiff-like clusters. The proportions of significantly upregulated genes (of all genes) for each KEGG pathway are shown. The *x*-axis represents the statistical significance expressed as −log10 (*p* value), and the *y*-axis indicates the pathways. (**D**) Fold-change expression levels of FoxO signaling pathway genes in the young-undiff-like and old-undiff-like clusters, each normalized to the expression levels in the young-undiff-like cluster, as determined via RNA sequencing. (**E**) Fold-change expression levels of lysosome-related genes in the young-undiff-like and old-undiff-like clusters, each normalized to the young-undiff-like cluster, as determined via RNA sequencing. (**F**) Functional enrichment hub gene analysis based on the young-undiff-like cluster. (**G**) Bar plot showing KEGG pathway enrichments of genes differentially expressed in the young-undiff-like cluster from (**F**). The *x*-axis represents the statistical significance expressed as −log10 (*p* value), and the *y*-axis indicates the pathways. Boxplot data are the first quartile, median, and third quartile. Values are means ± SEMs.

To investigate age-associated transcriptional differences among undifferentiated myoblasts, we performed DEG analysis using a distinct sample set derived from PCA clustering (Figure 3A). A comparison of the Young-Undiff-like and Old-Undiff-like clusters identified 1458 DEGs (DEG set 2), of which 825 were upregulated and 633 were downregulated in the young cluster (Figure 3B). To further explore functional differences, KEGG analysis of the DEGs (Figure 3C) revealed significant enrichment in pathways associated with FoxO signaling, cellular senescence, regulation of longevity, lysosomal function, focal adhesion, calcium signaling, ECM-receptor interaction, gap junctions, and amino acid biosynthesis. We focused on autophagy-related pathways due to autophagy’s critical role in muscle cell homeostasis and differentiation. Several genes in the FoxO signaling pathway, including *SOD2*, *PIK3CA*, *PIK3R1*, *PIK3CD*, *FOXO1*, *BCL2L11*, *FBXO32*, *FOXO4*, *SETD7*, *TGFBR2*, *BCL2L11*, and *MAPK11*, exhibited age-associated differential expression (Figure 3D and Figure S2A). Genes involved in the lysosomal pathway, such as *MAN2B1*, *GNPTAB*, *CTSF*, *ASAH1*, *GNS*, *CTSK*, *CTSL*, *FUCA2*, *SLC17A5*, *SCARB2*, and *BCL2L11*, were expressed at lower levels in Young-Undiff-like cells than in other groups (Figure 3E and Figure S2B). Evaluation of the longevity regulation pathway further highlighted age-dependent transcriptional differences; pathway genes were generally expressed at lower levels in young undifferentiated myoblasts than in old undifferentiated myoblasts (Figure S2D). Conversely, amino acid biosynthesis pathways were more highly expressed in young cells, suggesting increased metabolic activity. Genes such as *PC*, *PHGDH*, *BCAT1*, and *ARG2* were significantly upregulated in young samples (Figure S2E). GO analysis of BP identified further functional differences between age groups (Figure S2C). In young bovine undifferentiated cells, enriched processes included skeletal muscle tissue development, muscle contraction, muscle cell differentiation, and muscle structure development—each more active than in older cells. In contrast, older bovine undifferentiated cells showed significant enrichment in processes related to reduced fiber organization, actin filament depolymerization, and impaired cytoskeletal organization. These findings suggest a diminished cytoskeletal remodeling capacity in older cells, which may impair their differentiation potential. To identify the key regulatory genes, we performed hub gene analysis using the MCC algorithm in CytoHubba. This revealed two distinct functional networks (Figure 3F). The first network included genes associated with structural integrity and differentiation—such as *COL1A1*, *COL5A1*, *COL5A2*, *COL6A3*, *COL16A1*, *COL4A5*, *COL8A2*, *P3H1*, *P3H2*, *TGFB1*, *ATP2A1*, *MYOG*, *MYOD1*, and *PDGFRA*—primarily involved in extracellular matrix (ECM) remodeling and myogenic differentiation. The second network comprised genes related to metabolic and autophagic regulation, including *ASNS*, *ALDH1L2*, *PHGDH*, *PSPH*, *MTHFD2*, and *PSAT1*, which are associated with amino acid metabolism and energy homeostasis. Further GO analyses of these hub genes (Figure 3G) confirmed their involvement in age-specific regulatory mechanisms influencing myoblast differentiation and cellular homeostasis. Together, our findings reveal significant age-dependent transcriptional differences in undifferentiated bovine myoblasts. Young myoblasts exhibit greater metabolic activity, enhanced differentiation potential, and active ECM remodeling, whereas older myoblasts show increased expression of genes linked to cytoskeletal degradation and senescence. Moreover, hub gene analysis identified two distinct regulatory networks associated with structural integrity and metabolic regulation. These networks may serve as valuable targets when seeking to optimize muscle stem cell differentiation for cultured meat production.

### 3.4. Age-Specific Characterization of Differentiated Muscle Cells

**Figure 4:**
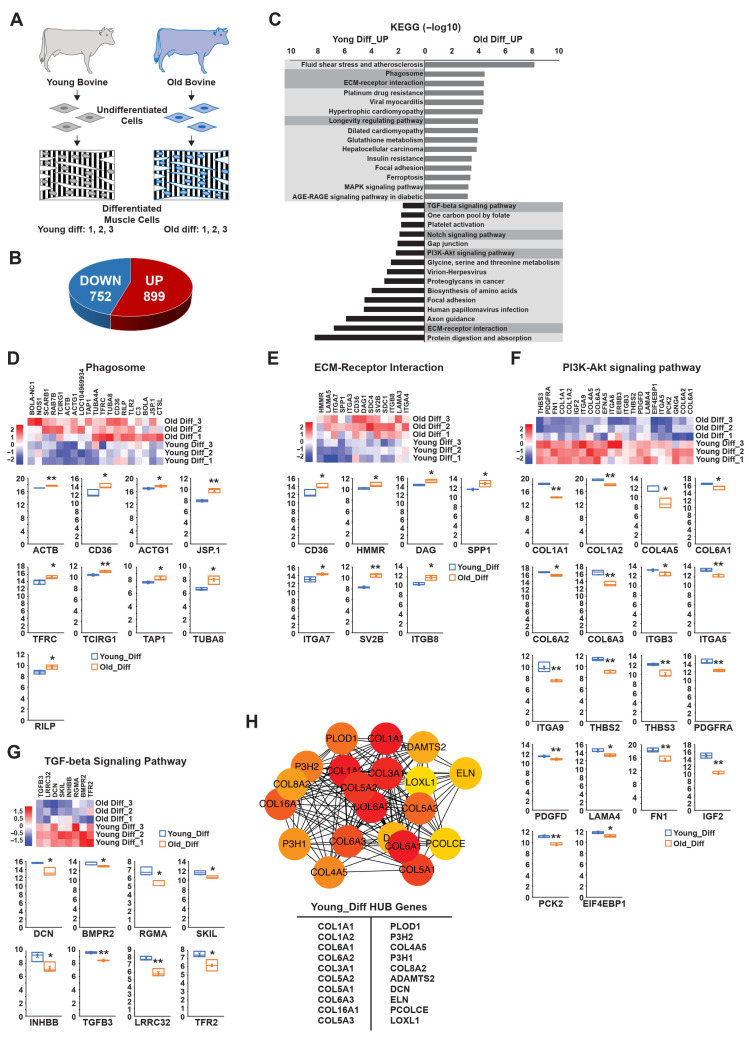
Age-related characterization of differentiated bovine muscle stem cells. (**A**) Schematic representation of young and old differentiated bovine cells used for transcriptomic analysis. (**B**) Pie chart displaying the numbers of genes with the greatest differences in upregulation and downregulation between the young-diff and old-diff clusters. (**C**) Bar plot showing KEGG pathway enrichments of genes differentially expressed in the young-diff and old-diff clusters. The proportions of significantly upregulated genes for each KEGG pathway are shown. The *x*-axis represents the statistical significance expressed as −log10 (*p* value), and the *y*-axis indicates the pathways. (**D**–**G**) Fold-change expression levels of genes related to: (**D**) phagosome signaling, (**E**) ECM-receptor interaction, (**F**) PI3K-Akt signaling, and (**G**) TGF-beta signaling in the young-diff and old-diff clusters, as determined via RNA sequencing. Bottom panels contain boxplots showing the expression levels of each gene and the differences between young and old muscle cells. (**H**) Functional enrichment hub gene analysis based on the young-diff cluster. Boxplot data are the first quartile, median, and third quartile. Values are means ± SEMs. * *p* < 0.05, ** *p* < 0.01.

Using the same analytical approach, we next investigated the transcriptional profiles of differentiated muscle cells from young and old bovines (Figure 4A). This analysis identified 1651 DEGs (DEG set 3), with 899 upregulated and 752 downregulated in the young group relative to the old group (Figure 4B). To classify these DEGs into functional categories, we performed KEGG pathway analysis, particularly focusing on pathways associated with autophagy and cellular regulation. In old bovines, notable pathway enrichment was observed for phagosome function, ECM-receptor interaction, and the longevity-regulating pathway (Figure 4C). Within the phagosome pathway, genes such as *ACTB*, *CD36*, *ACTG1*, *JSP.1*, *TFRC*, *TCIRG1*, *TAP1*, *TUBA8*, and *RILP* exhibited significantly increased expression levels in older bovines (Figure 4C,D). Similarly, within the ECM-receptor interaction pathway, genes including *CD36*, *HMMR*, *DAG*, *SPP1*, *ITGA7*, *SV2B*, and *ITGB8* were more highly expressed in old bovines than in young bovines (Figure 4C,E). Genes of the longevity-regulating pathway were also expressed at higher levels in the old group, consistent with previous findings suggesting an age-associated shift in cellular regulation (Figure S3B). In contrast, differentiated cells from young bovines exhibited increased expression in the TGF-beta, Notch, and PI3K-AKT signaling pathways (Figure 4C). Specifically, genes associated with the PI3K-Akt signaling pathway, including *COL4A5*, *ITGA9*, *THBS3*, and *IGF2*, were highly expressed in young muscle cells (Figure 4F). Similarly, within the TGF-beta signaling pathway, genes such as *DCN*, *RGMA*, and *LRRC32* showed elevated expression, reinforcing the role of TGF-beta in promoting myoblast differentiation (Figure 4G).

To further investigate the biological implications of these transcriptional differences, we subjected the BP associated with DEG set 3 to GO analysis (Figure S3A,C). In the young group, this analysis revealed significant enrichment of carbohydrate metabolism, suggesting that an enhanced metabolic state is associated with muscle differentiation. Conversely, downregulated DEGs were predominantly involved in carboxylic acid biosynthesis, regulation of membrane depolarization, and mitochondrial depolarization, all of which are linked to autophagic regulation. Notably, mitochondrial depolarization—closely associated with autophagic activity—was significantly higher in young bovines, supporting the hypothesis that autophagy is tightly linked to efficient muscle differentiation and energy metabolism. To identify key regulatory genes within this dataset, we subjected DEGs highly expressed in the young group to hub gene analysis. The top 20 hub genes were *COL1A1*, *COL1A2*, *COL6A1*, *COL6A2*, *COL3A1*, *COL5A2*, *COL5A1*, *COL6A3*, *COL16A1*, *COL5A3*, *PLOD1*, *P3H2*, *COL4A5*, *P3H1*, *COL8A2*, *ADAMTS2*, *DCN*, *ELN*, *PCOLCE*, and *LOXL1* (Figure 4H). These genes are primarily involved in ECM remodeling, muscle tissue organization, and structural integrity—essential functions for muscle development and performance. Further functional GO analysis of these hub genes (Figure S3D) revealed significant enrichment in protein digestion and absorption pathways, which are critical for myogenic differentiation and skeletal muscle maintenance. These findings align well with previous results obtained from undifferentiated cells and reinforce the importance of age-related molecular differences in myoblast function and differentiation potential.

### 3.5. Autophagy Maintains the Equilibrium of Myoblast Differentiation in a Myoblast Cell Line

**Figure 5:**
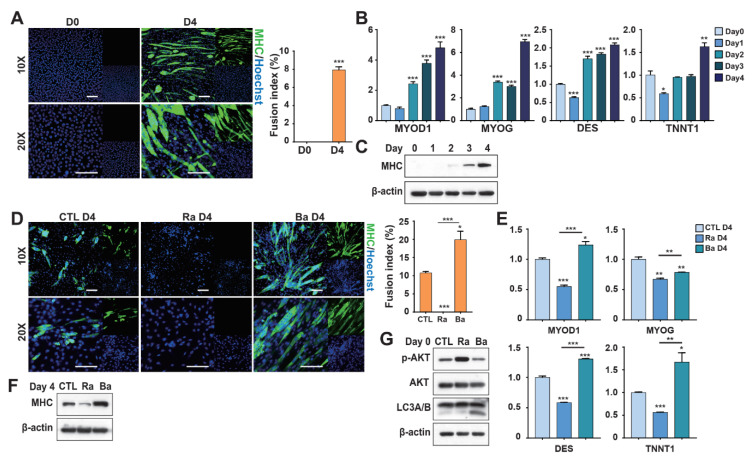
Autophagy preserves the balance of myoblast differentiation in a myoblast cell line. (**A**) MHC expression by C2C12 cells grown in differentiation medium (Day 4), revealed via immunocytochemical staining (scale bar: 100 μm). (**B**) Expression levels of myogenic transcription factors and canonical differentiation markers detected via RT-qPCR on days 0, 1, 2, 3, and 4 in differentiation medium. (**C**) MHC expression levels as proportions of total protein on days 0–4, as revealed by Western blotting. β-actin served as the internal control. (**D**) MHC expression in C2C12 cells treated with rapamycin (Ra) or Bafilomycin A1 (Ba) in growth medium, then switched to differentiation medium for 4 days (scale bar: 100 μm). (**E**) Expression of myogenic transcription factors and differentiation markers in Ra- or Ba-treated C2C12 cells, followed by differentiation for 4 days, as determined via RT-qPCR. (**F**) MHC protein levels in Ra- or Ba-treated C2C12 cells after 4 days in differentiation medium. (**G**) Protein levels of p-AKT, AKT, and LC3A/B in Ra- or Ba-treated C2C12 cells during growth medium incubation. Values are means ± SEMs (*n* = 3 independent replicates per group). * *p* < 0.05, ** *p* < 0.01, *** *p* < 0.001.

Transcriptomic analysis suggested that autophagy-related signaling plays a key regulatory role during myogenic differentiation. To investigate this mechanism, C2C12 mouse myoblasts were used as a model system. Differentiation induction for 4 days resulted in the formation of multinucleated myotubes and increased expression of myogenic markers, confirming successful myogenic differentiation (Figure 5A–C). To examine the role of autophagy, cells were pretreated with rapamycin (RAPA), an autophagy activator, or bafilomycin A1 (BafiA1), an autophagy inhibitor. These treatments produced opposite effects on differentiation: BafiA1 enhanced myotube formation, whereas RAPA maintained cells in a more undifferentiated state and prevented premature differentiation (Figure 5D–F). These changes were associated with modulation of the AKT signaling pathway, where RAPA increased AKT activation and BafiA1 suppressed it (Figure 5G). To determine whether these effects were stable, treated cells were passaged before differentiation induction. RAPA-treated cells partially recovered their differentiation capacity after one passage, although their differentiation efficiency remained lower than that of control cells (Figure S4A–D). In contrast, the differentiation-promoting effect of BafiA1 disappeared after passaging, with differentiation levels returning to those of untreated controls (Figure S4E–H). Together, these results indicate that autophagy modulation through AKT signaling dynamically regulates myoblast differentiation, but these regulatory effects are largely transient after cell passaging.

### 3.6. Autophagy Controls the Differentiation Potential of Young Bovine Myoblasts

**Figure 6:**
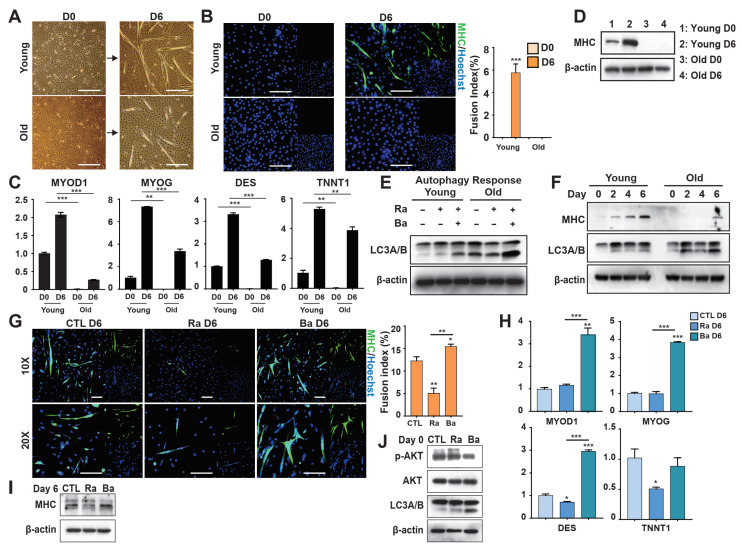
Autophagy regulates the myoblastic differentiation of bovine muscle stem cells. (**A**) Representative images of young and old bovine muscle stem cells cultured in differentiation medium on days 0 and 6 (scale bar: 100 μm). (**B**) MHC expression in young and old bovine muscle stem cells cultured in differentiation medium on day 6, as revealed by immunocytochemical staining (scale bar: 100 μm). (**C**) Expression levels of myogenic transcription factors and canonical differentiation markers in young and old bovine muscle stem cells on days 0 and 6 of differentiation, as determined via RT-qPCR. (**D**) MHC expression levels as a proportion of total protein in young and old bovine muscle stem cells on days 0 and 6 of differentiation, as revealed by Western blotting. β-actin served as the internal control. (**E**) LC3A/B expression levels as a proportion of total protein in young and old bovine muscle stem cells treated with Ra or Ra + Ba in growth medium, followed by a switch to differentiation medium on days 0, 2, 4, and 6. (**F**) Expression levels of MHC and LC3A/B as proportions of total protein in C2C12 cells treated with Ra or Ra + Ba in growth medium. (**G**) MHC expression by young bovine muscle stem cells treated with Ra or Ba in growth medium, followed by a switch to differentiation medium for 6 days, as revealed by immunocytochemical staining (scale bar: 100 μm). (**H**) Expression levels of myogenic transcription factors and canonical differentiation markers in young bovine muscle stem cells treated with Ra or Ba in growth medium, followed by 6 days in differentiation medium, as determined via RT-qPCR. (**I**) MHC expression levels in young bovine muscle stem cells treated with Ra or Ba in growth medium, followed by 6 days in differentiation medium. (**J**) Expression levels of p-AKT, AKT, and LC3A/B as proportions of total protein in young bovine muscle stem cells treated with Ra or Ba in growth medium. Values are means ± SEMs (*n* = 3 independent replicates per group). * *p* < 0.05, ** *p* < 0.01, *** *p* < 0.001.

The results obtained using the C2C12 myoblast cell line suggest that AKT-related autophagic signaling plays an important role in regulating myogenic differentiation. To further investigate this mechanism, primary bovine myoblasts were isolated and cultured, and a differentiation protocol was successfully established. After 6 days of culture, the cells formed well-defined muscle structures (Figure 6A). Notably, fetal-derived (Young) muscle cells exhibited significantly greater differentiation capacity than adult-derived (Old) cells, as evidenced by increased myotube formation and a higher fusion index (Figure 6A,B). Consistently, the expression levels of myogenic differentiation markers were substantially higher in Young samples than in Old samples (Figure 6C,D), indicating stronger differentiation potential in Young myoblasts. Interestingly, autophagic activity was significantly lower in Young samples than in Old samples during differentiation (Figure 6E,F), suggesting that reduced autophagy is associated with enhanced differentiation capacity. These observations indicate that autophagy may negatively regulate myogenic differentiation and that this regulatory effect differs across developmental stages. To further validate the functional role of autophagy, Young-derived cells were treated with BafiA1 or RAPA. The results were consistent with those obtained in C2C12 cells: BafiA1 enhanced myogenic differentiation, whereas RAPA suppressed differentiation and maintained cells in a more undifferentiated state (Figure 6G–J). We next examined whether these regulatory effects persisted after cell passaging. Young cells pre-treated with RAPA (Ra P + 1) continued to show reduced differentiation capacity after passaging, accompanied by sustained activation of AKT signaling (Figure S5A–D). In contrast, cells pre-treated with BafiA1 (Ba P + 1) displayed diminished differentiation compared with their initial response, indicating that the pro-differentiation effects of autophagy inhibition were not long-lasting (Figure S5E–H). Together, these findings indicate that Young-derived myoblasts possess greater differentiation potential than Old-derived cells and that autophagy negatively regulates this process. Moreover, modulation of autophagy through AKT signaling dynamically regulates differentiation, but these effects are largely transient after passaging.

## 4. Discussion

Cultured meat production is an alternative to conventional livestock farming. It has been suggested that this approach may reduce greenhouse gas emissions, require less land and water, and potentially improve food safety due to controlled production conditions. The need for animal slaughter could be substantially reduced, which may help us to address some ethical concerns among consumers, although ethical issues related to cultured meat remain under discussion. The use of antibiotics in cultured meat production has been proposed to be potentially reduced compared with conventional livestock systems, which may help mitigate concerns related to antibiotic resistance [33]. However, challenges persist regarding scalability, cost-effectiveness, and optimization of muscle stem cell differentiation [34]. Bovine skeletal muscle stem cell differentiation is governed by several factors, including key signaling pathways (e.g., Notch, Wnt, TGF-β and PI3K-AKT), transcription factors (e.g., MyoD, Myf5, Myogenin), and autophagy. These components control satellite cell activation, proliferation, and differentiation [35,36,37,38]. Transcriptomic analyses provide valuable insights into these regulatory processes by identifying gene expression changes that guide myogenesis and cellular commitment. A full understanding of these mechanisms is essential to enhance differentiation efficiency and optimize the large-scale production of cultured meat [32,39].

In this study, we used RNA sequencing to define the transcriptomic landscapes of bovine muscle stem cells at different developmental stages (Figure S6). Our results demonstrate that age significantly influences gene expression dynamics during differentiation. Fetal-derived bovine myoblasts showed greater transcriptional stability and higher differentiation potential than adult-derived cells. Specifically, young samples exhibited consistency between undifferentiated and differentiated states, whereas older samples displayed greater variability, supporting previous reports that aging disrupts gene regulation during myogenesis [40]. KEGG pathway analysis revealed that the Rap1 and AMPK signaling pathways are among the significantly enriched pathways in our differentially expressed genes and are therefore highlighted as key regulators of myogenic differentiation. The Rap1 pathway is known to control cell proliferation, adhesion, and migration, whereas the AMPK pathway regulates energy metabolism and cellular stress responses [41,42]. Other pathways, such as calcium signaling or proteoglycans in cancer, were also enriched in our analysis; however, we focused on Rap1 and AMPK signaling because these pathways showed higher enrichment scores and are well-established regulators of myogenic differentiation. The upregulation of these pathways in differentiated cells highlights their functional roles in muscle development and metabolic adaptation. In contrast, pathways related to p53 signaling, cellular senescence, and cell cycle regulation were more prominent in undifferentiated myoblasts, suggesting their involvement in maintaining proliferative capacity and preventing premature differentiation [43,44]. Functional enrichment analyses further supported this distinction, with genes associated with muscle system processes, contraction, and differentiation enriched in differentiated cells, whereas pathways related to DNA replication and cell proliferation were more characteristic of undifferentiated cells. These patterns indicate a transcriptional shift from proliferative programs toward differentiation-associated functions during myogenesis. Notably, young bovine muscle stem cells exhibited a more defined transcriptional transition during differentiation, whereas cells derived from older bovines showed greater heterogeneity. This observation suggests that aging may impair the coordinated transcriptional activation required for efficient myoblast differentiation.

Our findings indicate that aging is associated with distinct transcriptional programs in undifferentiated myoblasts, particularly in pathways related to cellular aging, metabolism, and cytoskeletal regulation. The enrichment of FoxO signaling, cellular senescence, and longevity-related pathways in older cells suggests an increased activation of stress-response and aging-associated regulatory mechanisms. The FoxO pathway is a well-established regulator of cellular aging and autophagy and plays a pivotal role in muscle maintenance, with FOXO3 and FOXO4 serving as key actors [45,46]. Additionally, lysosome-related genes were upregulated in old undifferentiated cells compared to young cells, suggesting a potential compensatory increase in lysosomal activity with age, which may reflect altered autophagic regulation in older cells [47,48]. In contrast, young myoblasts showed stronger enrichment of metabolic and differentiation-associated programs, consistent with previous studies demonstrating that amino acid metabolism supports muscle cell growth and differentiation [49]. Consistent with this interpretation, genes involved in muscle development and differentiation were more prominently represented in young cells, whereas aging appeared to be associated with reduced cytoskeletal organization, a process known to impair myogenic differentiation capacity [50].

Differentiated myoblasts also exhibited clear age-related transcriptional differences, suggesting that aging influences regulatory programs active during later stages of myogenesis. Pathways associated with extracellular matrix (ECM) interaction and longevity regulation were more prominent in older cells, whereas signaling pathways related to myogenic differentiation, including TGF-β, Notch, and PI3K-Akt, were more active in young differentiated cells. The ECM–receptor interaction pathway plays a crucial role in muscle regeneration and aging, as impaired ECM remodeling can disrupt muscle architecture and signaling [51]. The increased representation of longevity-related pathways in older cells may reflect a compensatory response to cellular stress and aging-associated damage [52]. Functional enrichment patterns further suggest that aging is accompanied by mitochondrial dysfunction and oxidative stress, whereas younger myoblasts maintain stronger metabolic activity, supporting more efficient proliferation and differentiation potential [53,54]. Genes associated with ECM organization, particularly collagen family members, were prominently represented among age-related regulators, consistent with previous studies showing that ECM composition dynamically changes during myogenesis and influences cytoskeletal organization and intracellular signaling [55]. In particular, collagen family members are known to contribute to muscle mechanical stability and aging-related structural remodeling, highlighting their potential role in sustaining differentiation efficiency [56]. In contrast, the higher activity of TGF-β–related regulatory genes in young muscle cells may support efficient myoblast proliferation and differentiation. Notably, TGF-β signaling exerts context-dependent effects during muscle regeneration: moderate activation promotes myogenic progression, whereas excessive signaling can inhibit regeneration and promote fibrosis [57]. Together, these observations suggest that age-related alterations in ECM organization and signaling balance may contribute to the reduced differentiation efficiency observed in older muscle stem cells.

Our findings further highlight the important role of autophagy in regulating myogenic differentiation, particularly through its interaction with the AKT signaling pathway, a central regulator of cellular metabolism, proliferation, and differentiation. Autophagy contributes to cellular homeostasis by maintaining energy balance and protein turnover and has been widely implicated in muscle development and regeneration [58]. Previous studies have also demonstrated crosstalk between AKT signaling and autophagic regulation during myogenesis, indicating that coordinated activity between these pathways is essential for proper muscle cell differentiation [59,60]. In the present study, pharmacological modulation of autophagy in the C2C12 myoblast model further supported this regulatory relationship. Inhibition of autophagy promoted myogenic differentiation, whereas autophagy activation suppressed differentiation and maintained cells in a less differentiated state, consistent with the concept that excessive autophagic activity may disrupt cellular homeostasis and limit myogenic progression. These findings suggest that autophagy exerts a dose-dependent influence on muscle differentiation, where moderate autophagic activity supports cellular remodeling while excessive activation may degrade essential components required for myogenic commitment. Importantly, the regulatory effects of autophagy appeared to be dynamic and gradually diminished over successive passages, suggesting that autophagic signaling is influenced by cellular status and culture conditions. Our results also indicate that age-related differences in autophagic regulation may contribute to the distinct differentiation capacities observed between young and old bovine muscle stem cells. Cells derived from younger donors displayed stronger differentiation potential with relatively lower autophagic activity, whereas cells from older donors showed elevated autophagic activity accompanied by reduced differentiation efficiency. Together, these observations suggest that balanced AKT–autophagy signaling is critical for maintaining efficient myogenic differentiation. Understanding how this regulatory axis can be modulated may provide valuable insights for improving the stability and efficiency of muscle cell differentiation in cultured meat production systems.

This study explored the role of autophagy in muscle differentiation, with a particular focus on the dynamic interplay between autophagy and the AKT signaling pathway. The findings demonstrate that the level of autophagy influences myogenic differentiation: low-level autophagy promotes differentiation, whereas high-level autophagy is suppressive. However, this study has several limitations. First, it primarily examines the short-term effects of autophagic regulation on muscle differentiation, rather than long-term outcomes—particularly those associated with repeated cell passaging [61]. Second, this research utilizes both mouse myoblast (C2C12) and bovine muscle stem cells, which may exhibit species-specific differences that limit the generalizability of the findings. Cross-species validation studies are therefore needed to determine whether autophagic regulation of myogenesis is conserved across species. Finally, although key signaling pathways, including AKT signaling, autophagy, and energy metabolism, were identified as central regulators of myoblast differentiation, the precise molecular mechanisms underlying their interactions remain to be fully elucidated, particularly the molecular interaction between AKT signaling and autophagy regulators such as FOXO3. Future studies integrating additional omics approaches, such as proteomics and metabolomics, will be valuable to further dissect these regulatory networks. Looking ahead, further research should evaluate the long-term effects of autophagic regulation, especially during extended cell culture and passaging. Additionally, cross-species investigations could confirm the universality of this regulatory mechanism, and in-depth mechanistic studies may provide valuable insights into the autophagy–AKT signaling axis. Such research could uncover new targets and inform novel strategies to enhance muscle differentiation efficiency [62].

## 5. Conclusions

This study demonstrates that the developmental stage of bovine muscle tissue donors significantly influences muscle stem cell differentiation potential. Cells derived from young bovine exhibit greater transcriptional stability and superior differentiation capacity compared to those from older bovine. Key signaling pathways, including Rap1, AMPK, TGF-β, and Notch, are differentially expressed and contribute to these age-dependent differences. Functional experiments further confirmed that differentiation is dynamically regulated via the AKT–autophagy signaling pathway, with AKT phosphorylation serving as a key modulator: moderate autophagy promotes differentiation, whereas excessive autophagy inhibits it. These findings highlight the advantage of using young donor tissues and suggest that precise modulation of autophagy could optimize myogenic differentiation, providing a strategic foundation for efficient cultured meat production.

## Data Availability

The data presented in this study are available on request from the corresponding author.

## Supplementary Materials

The following supporting information can be downloaded at https://www.mdpi.com/article/10.3390/foods15061074/s1. Figure S1: Supplementary Figure S1 (related to Figure 1); Figure S2: Supplementary Figure S2 (related to Figure 3); Figure S3: Supplementary Figure S3 (related to Figure 4); Figure S4: Supplementary Figure S4 (related to Figure 5); Figure S5: Supplementary Figure S5 (related to Figure 6); Figure S6: Schematic of bovine skeletal muscle transcriptomic analysis to support improvements in cultured meat production; Figure S7: Supplemental western blots; Table S1: Primers used in RT-qPCR; Table S2: Antibodies used in western blotting and immunofluorescence assay.
