# Supplementary material for: The Transcriptomic Landscape and Regulatory Signaling Features of Bovine Skeletal Muscle Cells Used for Cultured Meat Production
Source: Foods. 2026 Mar 19;15(6):1074. doi: 10.3390/foods15061074 (PMC13025825; doi:10.3390/foods15061074)
Supplement: Supplementary file 1 [file foods-15-01074-s001.zip › foods-4177128-supplementary.pdf]

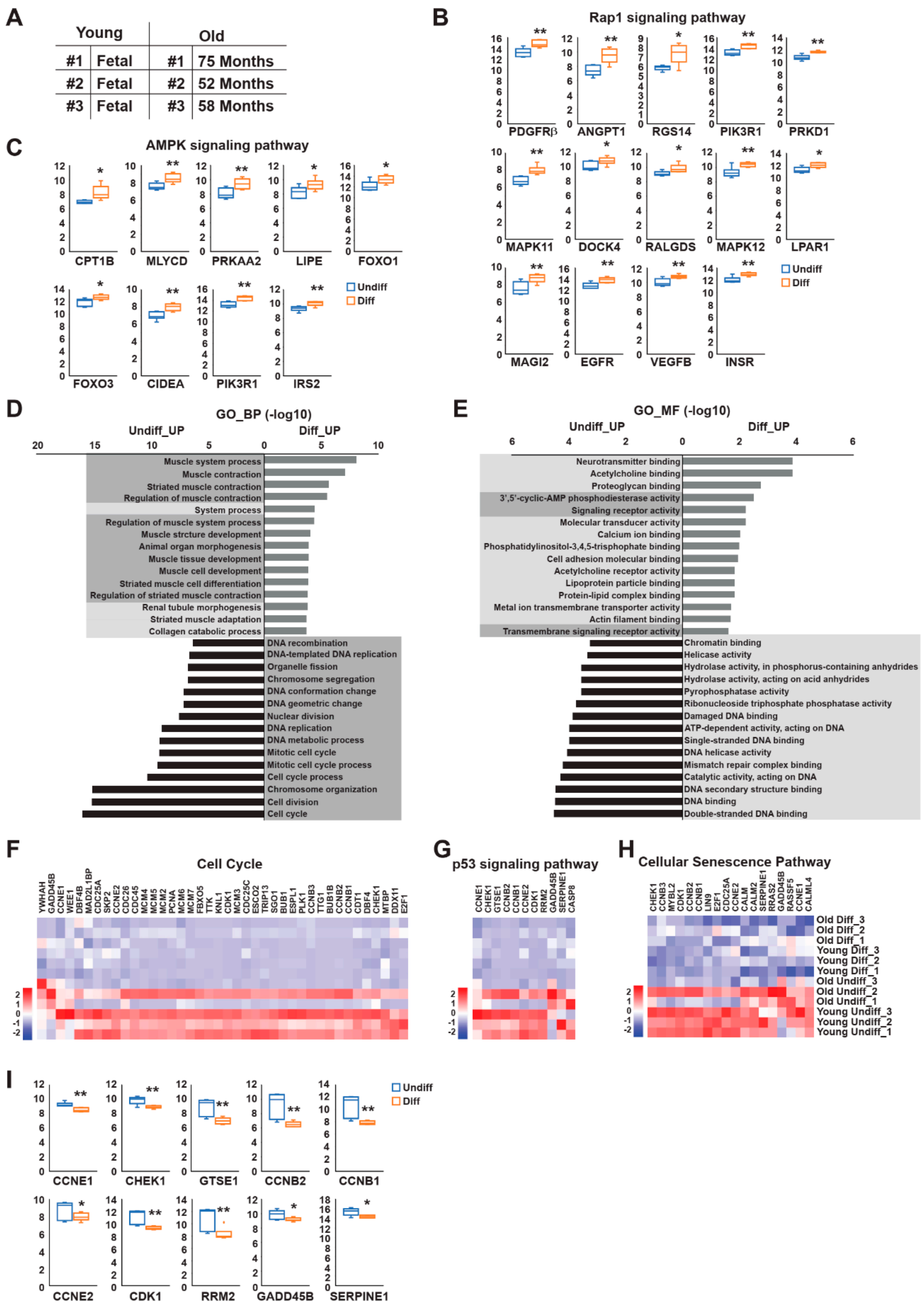

Figure S1. (A) The bovine samples subjected to transcriptomic analysis. (B) Expression levels of Rap1 signaling pathway genes in undifferentiated and differentiated cells, each normalized to the levels in undifferentiated cells, as determined via RNA sequencing related to Figure 1F. (C) Expression levels of AMPK signaling pathway genes in undifferentiated and differentiated cells, each normalized to those of undifferentiated cells, as determined via RNA sequencing related to Figure 1G. (D) Enriched biological process Gene Ontology

(GO_BP) terms and *p*-values comparing undifferentiated and differentiated muscle cells. (E) Enriched molecular function Gene Ontology (GO_MF) terms and p-values comparing undifferentiated and differentiated muscle cells. (F–H) Heatmaps showing the mean expression levels of genes involved in: (F) Cell cycle regulation, (G) p53 signaling pathway, and (H) cellular senescence pathway. Genes (columns) and samples (rows) are clustered by fold changes, ranging from –2 to 2. (I) Boxplot showing expression levels of genes in the p53 signaling pathway in undifferentiated and differentiated muscle cells. Boxplot data are the first quartile, median, and third quartile. Values are means ± SEMs. * $p < 0.05$, ** $p < 0.01$.

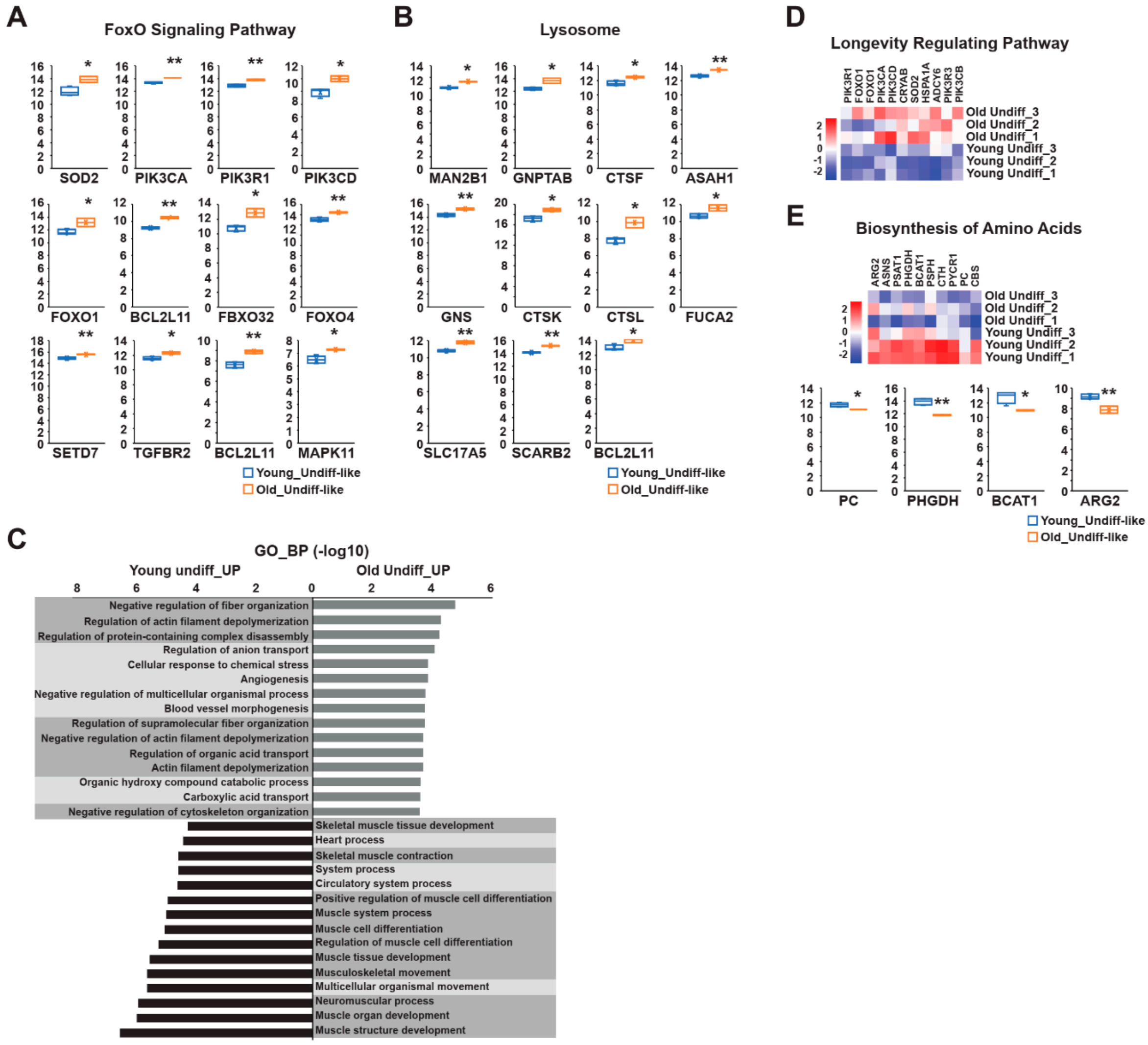

Figure S2. (A) Boxplots showing FoxO signaling pathway genes expression in the young-undiff-like and old-undiff-like clusters, as determined via RNA sequencing related to Figure 3D. (B) Boxplots showing lysosome-related genes expression in the young-undiff-like and old-undiff-like clusters, as determined via RNA sequencing related to Figure 3E. (C) Enriched biological process Gene Ontology (GO_BP) terms and *p*-values comparing undifferentiated young-like and old muscle cells. (D) Heatmap showing mean expression levels of genes involved in the longevity regulation pathway. (E) Heatmap showing mean expression levels of genes associated with amino acid biosynthesis. Bottom panels show boxplots of all genes with significant expression differences between undifferentiated young and old muscle cells. Boxplot data are the first quartile, median, and third quartile. Values are means ± SEMs. * $p < 0.05$, ** $p < 0.01$.

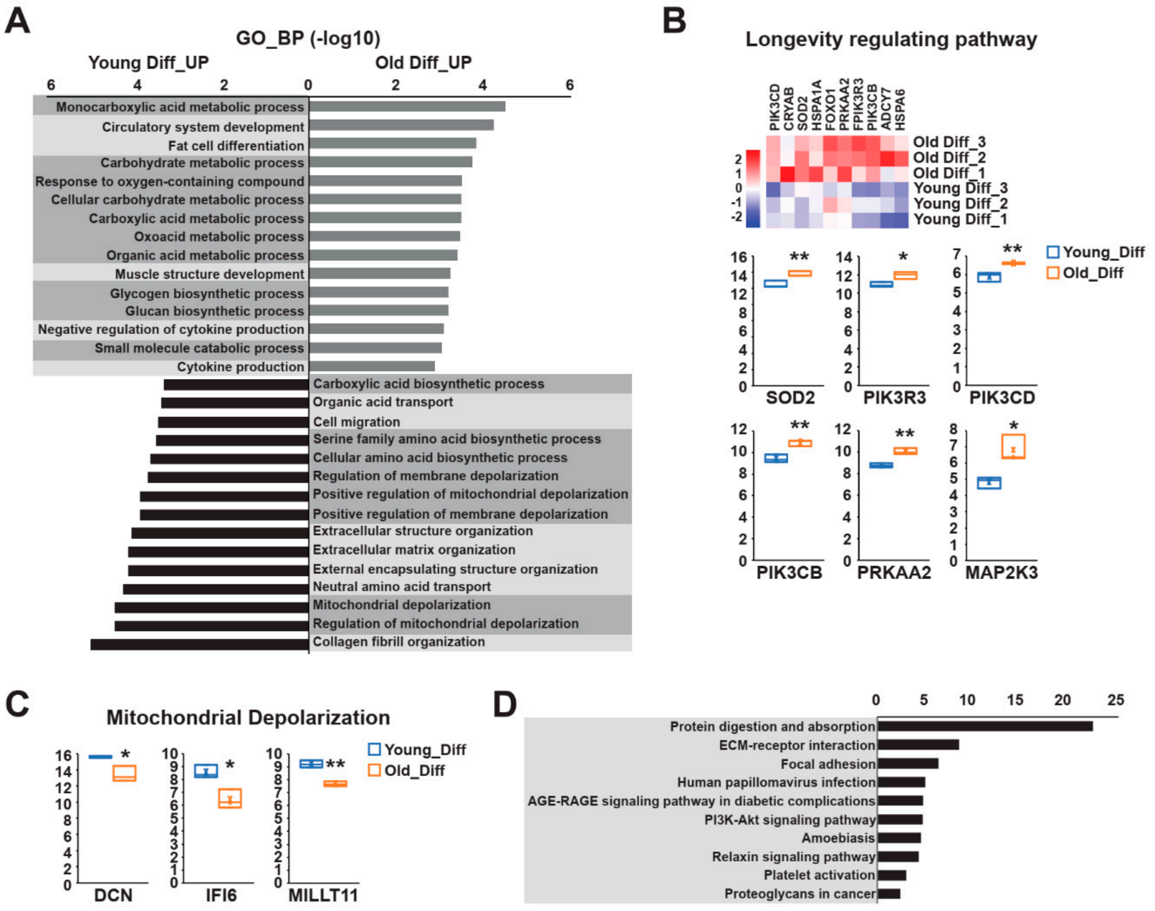

Figure S3. (A) Enriched biological process Gene Ontology (GO_BP) terms and *p*-values comparing differentiated cells from young and old bovine muscle samples. (B) Heatmap of the mean expression levels of genes involved in longevity regulation. Lower panels include boxplots showing expression levels of genes with distinct differences between differentiated young and old bovine muscle cells. (C) Boxplot showing expression level differences for genes involved in mitochondrial depolarization between differentiated young and old bovine muscle cells. (D) KEGG analysis for hub genes differentially expressed in bovine muscle cells differentiated from young and old samples. Boxplot data are the first quartile, median, and third quartile. Values are means ± SEMs. * $p < 0.05$, ** $p < 0.01$.

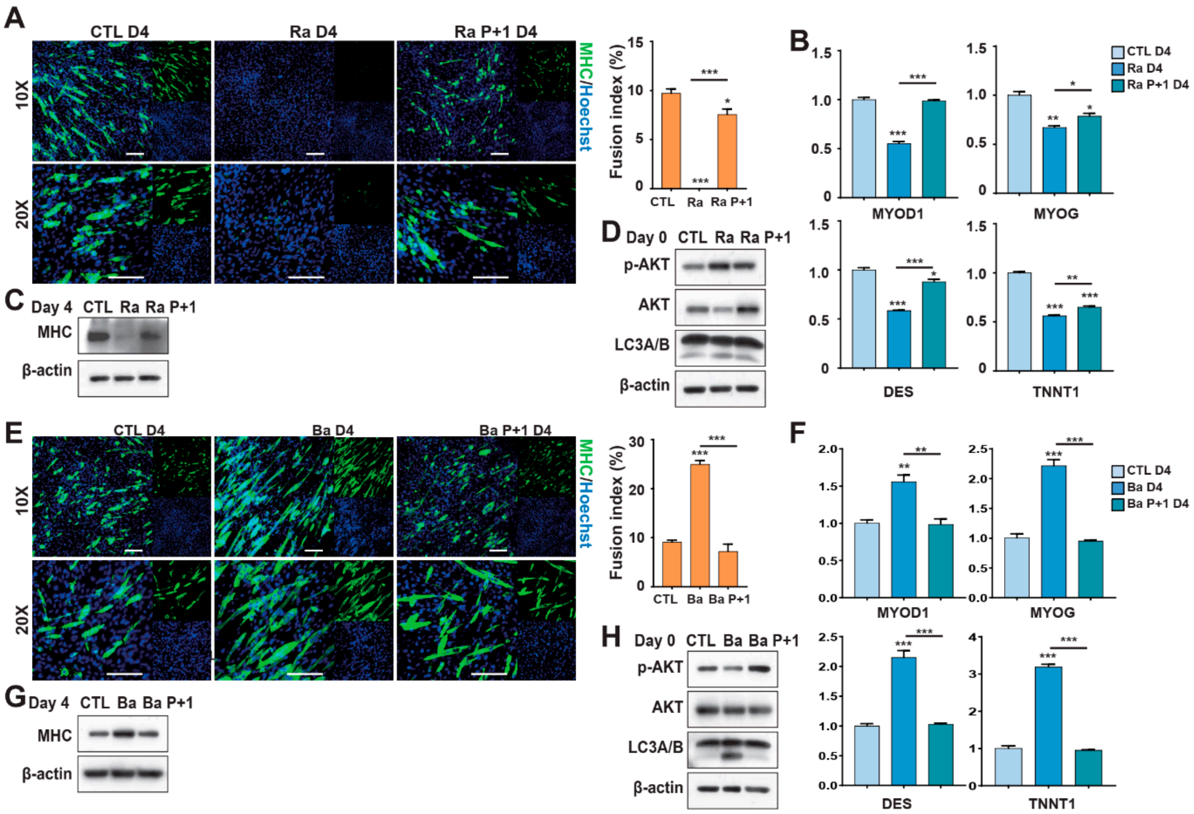

Figure S4. (A) MHC expression by C2C12 cells treated with Ra in growth medium, followed by one passage (Ra P+1) in cell culture and a switch to differentiation medium for 4 days, as revealed by immunocytochemical staining (scale bar: 100 μm). (B) Expression levels of myogenic transcription factors and canonical differentiation markers in Ra P+1 cells after 4 days of differentiation, as determined via RT-qPCR. (C) MHC protein levels in Ra P+1 cells after differentiation, as a proportion of total protein. (D) Protein levels of p-AKT, AKT, and LC3A/B in Ra P+1 cells. (E) MHC expression in Ba P+1 cells after differentiation, as revealed by immunocytochemical staining (scale bar: 100 μm). (F) Expression of myogenic transcription factors and differentiation markers in Ba P+1 cells after 4 days in differentiation medium, as determined via RT-qPCR. (G) MHC levels as a proportion of total protein in Ba P+1 cells. (H) Protein levels of p-AKT, AKT, and LC3A/B in Ba P+1 cells after one passage in culture. Values are means ± SEMs (n = 3 independent replicates per group). * $p < 0.05$, ** $p < 0.01$, *** $p < 0.001$.

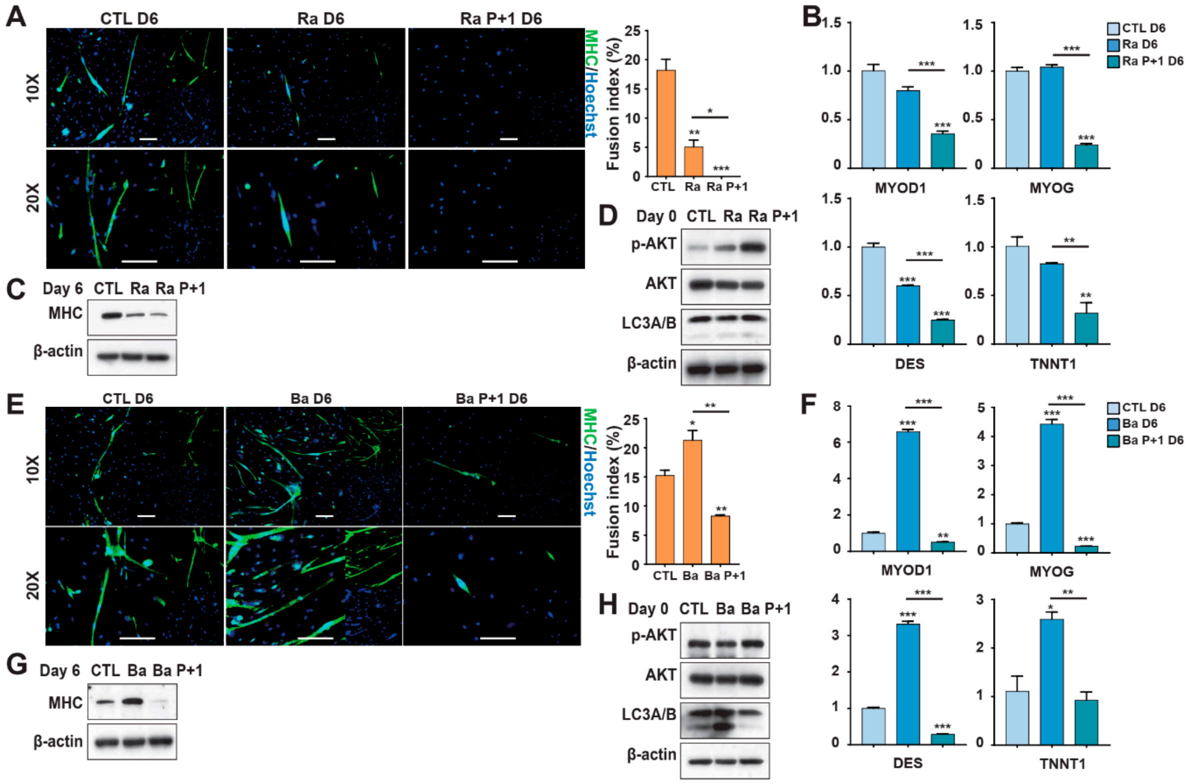

Figure S5. (A) MHC expression in young bovine muscle stem cells treated with Ra in growth medium, followed by one passage (Ra P+1) and 6 days in differentiation medium, as revealed by immunocytochemical staining (scale bar: 100 μm). (B) Expression levels of myogenic transcription factors and canonical differentiation markers in young bovine muscle stem cells treated with Ra in growth medium, then passaged once (Ra P+1) and cultured for 6 days in differentiation medium, as determined via RT-qPCR. (C) MHC expression levels as proportions of total protein in young bovine muscle stem cells treated with Ra in growth medium, followed by one passage (Ra P+1) and 6 days in differentiation medium. (D) Expression levels of p-AKT, AKT, and LC3A/B as proportions of total protein in young bovine muscle stem cells treated with Ra in growth medium, followed by one passage (Ra P+1) in cell culture. (E) MHC expression in young bovine muscle stem cells treated with Ba in growth medium, followed by one passage (Ba P+1) and 6 days in differentiation medium, as revealed by immunocytochemical staining (scale bar: 100 μm). (F) Expression levels of myogenic transcription factors and canonical differentiation markers in young bovine muscle stem cells treated with Ba in growth medium, then passaged once (Ba P+1) and cultured in differentiation medium for 6 days, as determined via RT-qPCR. (G) MHC expression levels as proportions of total protein in young bovine muscle stem cells treated with Ba in growth medium, followed by one passage (Ba P+1) and 6 days in differentiation medium. (H) Expression levels of p-AKT, AKT, and LC3A/B as proportions of total protein in young bovine muscle stem cells treated with Ba in growth medium, followed by one passage (Ba P+1) in cell

culture. Values are means ± SEMs (n = 3 independent replicates per group). * $p < 0.05$, ** $p < 0.01$, *** $p < 0.001$.

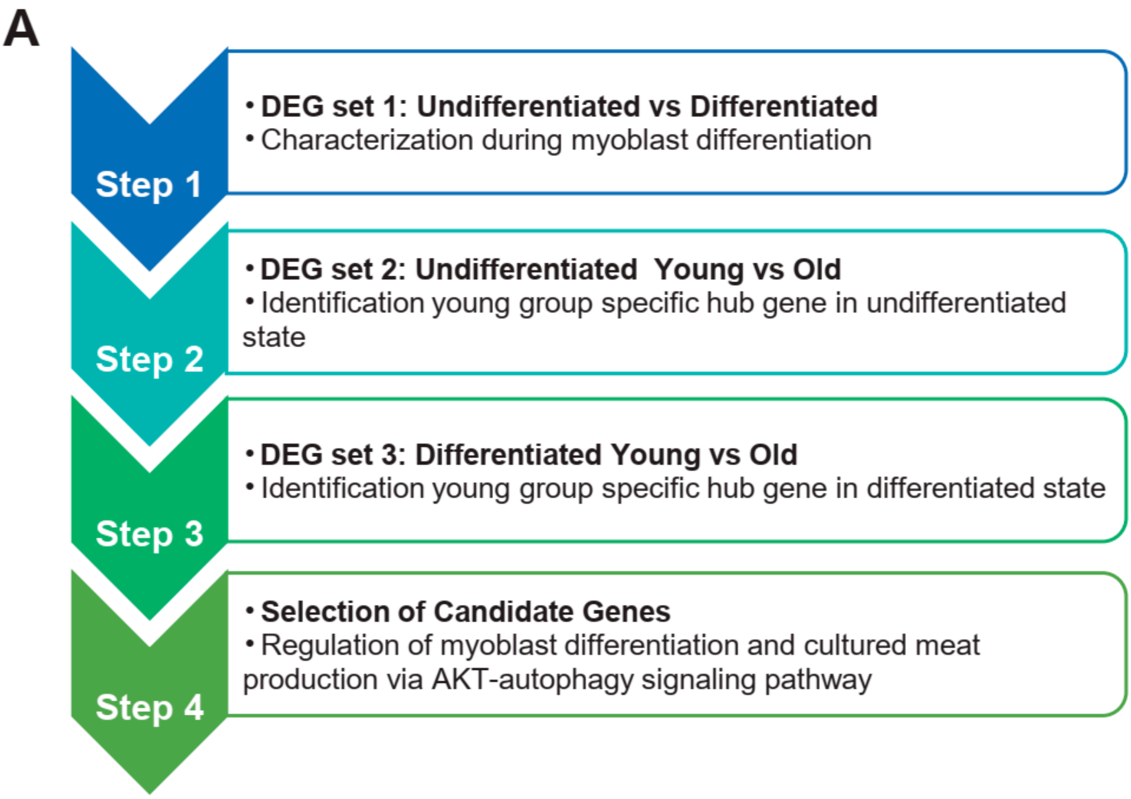

Figure S6. (A) Schematic of bovine skeletal muscle transcriptomic analysis to support improvements in cultured meat production.

Table S1. Primers used in RT-qPCR.

Gene	5'-3'
mGAPDH-F	AACTTTGGCATTGTGGAAGG
mGAPDH-R	ACACATTGGGGGTAGGAACA
mTNNT1-F	CTGTGGTGCCTCCTTTGATTC
mTNNT1-R	TGCGGTCTTTTAGTGCAATGAG
mMYOG-F	CTAAAGTGGAGATCCTGCGC
mMYOG-R	GACCGAACTCCAGTGCATTG
mDES-F	CAAGCAGGAGATGATGGAATACC
mDES-R	CATCTCATCCTTTAGGTGTCGG
mMYOD-F	CCAACTGCTCTGATGGCATG
mMYOD-R	GTGCATCTGCCAAAAGCAGC
bGAPDH-F	CGACTTCAACAGCGACACTCAC
bGAPDH-R	CCCTGTTGCTGTAGCCGAATTC
bTNNT1-F	CCTCTGATCCCGCCAAAGAT
bTNNT1-R	GGTCCTTTTCCATGCGCTTC
bMYOG-F	GCGCAGACTCAAGAAGGTGA
bMYOG-R	TGCAGGCGCTCTATGTACTG
bDES-F	GGAAGCCGAGGAATGGTACA
bDES-R	TCGATCTCGCAGGTGTAGGA
bMYOD-F	CGACGGCATGATGGACTACA
bMYOD-R	GTAAGTGCGGTCGTAGCAGT

Table S2. Antibodies used in western blotting and immunofluorescence assay.

Antibody	Company	Cat #	RRID
**Unconjugated 1th antibody**			
MHC	DSHB	MF 20	AB_2147781
Phospho-Akt (Ser473) (D9E) XP® Rabbit mAb	CST	4060	AB_2315049
Akt Antibody	CST	9272	AB_329827
LC3A/B Antibody	CST	4108	AB_2137703
β-actin	Santacruz	sc-47778	AB_626632
**Conjugated 2nd antibody**			
Alexa Fluor 488 donkey anti-Mouse IgG (H+L)	Invitrogen	A-21202	AB_141607
**Unconjugated 2nd antibody**			
Goat Anti-Mouse IgG (H+L) HRP Conjugate, human IgG (H+L) adsorbed	BIO-RAD	BR1706516	AB_2921252
Goat Anti-Rabbit IgG (H+L) HRP Conjugate, human IgG adsorbed	BIO-RAD	BR1706515	AB_11125142
